# Seed function in space and time: how spatial transcriptomics completes the picture

**DOI:** 10.3389/fpls.2026.1864389

**Published:** 2026-07-07

**Authors:** Tori Millsteed, Robert J. Henry

**Affiliations:** 1 Queensland Alliance for Agriculture and Food Innovation (QAAFI), University of Queensland, Brisbane, QLD, Australia; 2 Australian Research Council (ARC), Centre of Excellence for Plant Success in Nature and Agriculture, University of Queensland, Brisbane, QLD, Australia; 3 VinUni Big Data Research Institute, VinUniversity, Hanoi, Vietnam

**Keywords:** cell-type, embryo, endosperm, seed coat, tissue-type

## Abstract

Spatiotemporal gene expression is the underlying determinant of how living things grow and function, including the processes of seed development and germination. Understanding how these regulatory pathways work in space and time is critical to harnessing the functional diversity of the components of the seed and enhancing beneficial traits. The recent development of spatial transcriptomics technologies has greatly added to the knowledge of gene expression within plant and animal tissues but has only been applied in a handful of studies to seeds. Here we conduct a review of the literature on spatial omics research in developing and germinating seeds of wheat, barley, maize, rice, wild soybean and Arabidopsis, highlighting the major contributions to the field and the challenges faced. We outline how spatial transcriptomics has significantly improved the scale and resolution of gene expression analyses, revealing how the transcriptome explains the complete tissue architecture of the seed. This has resulted in the discovery of novel cell-subtypes, spatiotemporal gene expression gradients, and coordinated nutrient transport across tissues, completing the picture of seed composition in greater detail than could previously be achieved.

## Introduction

**Figure 1:**
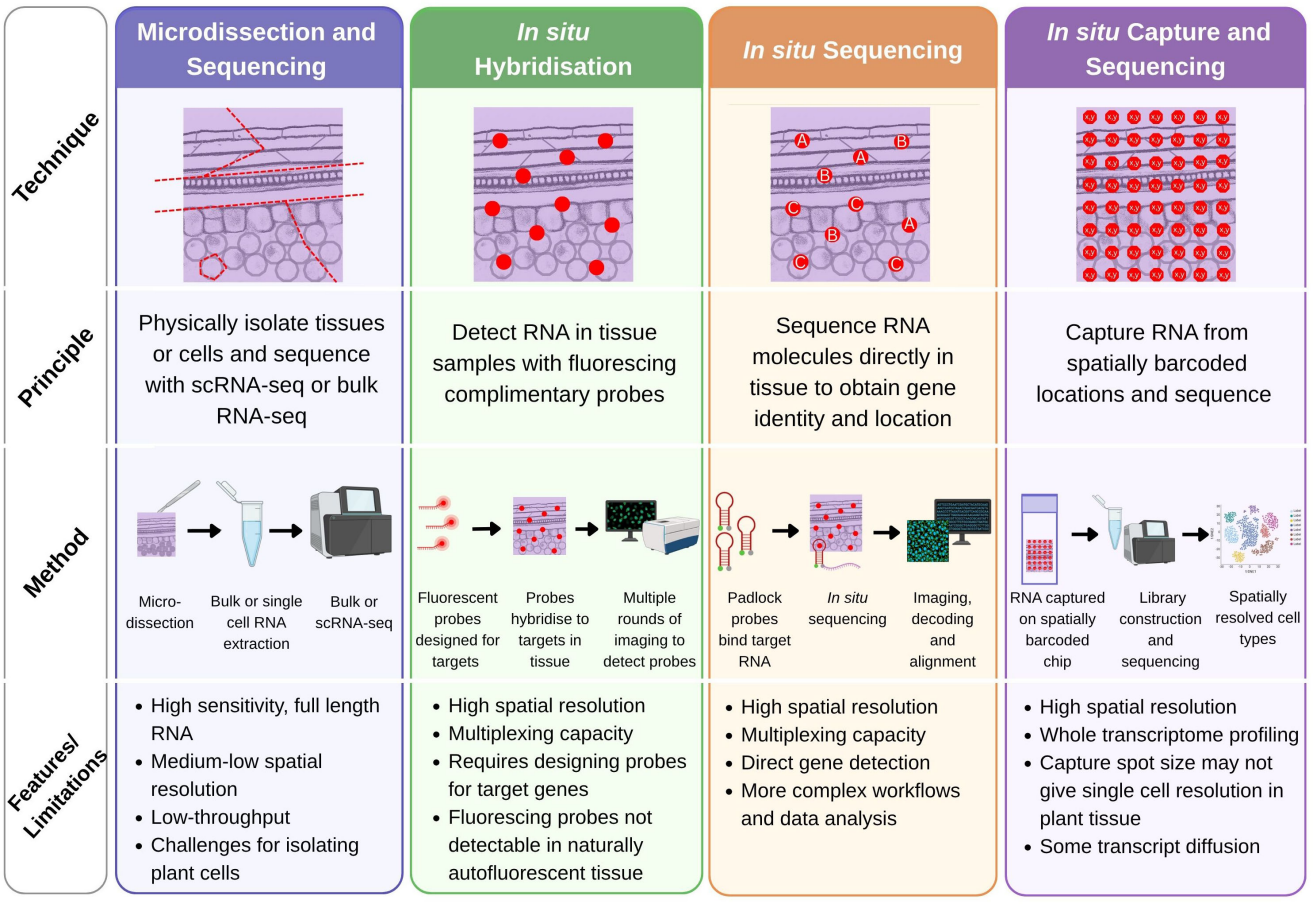
Overview of spatial transcriptomics techniques available including microdissection and sequencing, *in situ* hybridisation, *in situ* sequencing and *in situ* capture and sequencing. Describes the general principle, simplified methods and key features and limitations. (Created in BioRender. Millsteed et al. (2026) *https://BioRender.com/3zizr15*).

Seeds are the critical containers of plant life that mark the beginning of the lifecycle. They facilitate the genetic dispersal of angiosperms and provide an energy source to the germinating seedling, dictating the success of the subsequent plant (Chaudhury et al., 2001). Additionally, seeds have significant agricultural value as a food product, and important relevance to environmental conservation efforts (Dwivedi et al., 2021; Theissinger et al., 2023). Therefore, understanding the spatiotemporal gene expression that drives seed development and germination is fundamental to advancing crop improvement and ecological research. The complex structure of seeds, in most angiosperms, begins with double fertilisation and formation of the endosperm and embryo, the two main tissue types (Chaudhury et al., 2001). In monocots such as cereal grains, development then occurs through sequential stages of nuclear division, elongation and cellularisation, and accumulation of storage reserves such as starch and proteins in the large endosperm tissue, which provide energy to the embryo at germination. This process takes place within the third, protective tissue layer, the seed coat, which originates as the maternal ovule at fertilisation and is fused with the external pericarp cells (Shewry et al., 2012). In dicots such as *Arabidopsis thaliana*, the endosperm develops early and is then consumed by the growing embryo throughout development, resulting in a mature seed consisting of the large embryo, a thin remaining endosperm layer and the outermost seed coat layers (Nowack et al, 2010). As different tissues contribute differently to the functional and nutritional properties of seeds, understanding the genetic regulators of seed function within the complex tissue architecture would facilitate more targeted genetic research (Dwivedi et al., 2021). In recent decades, spatial gene expression has been studied through manual microdissection of cells and bulk or single-cell RNA sequencing. However, these techniques lack the fine resolution to analyse gene expression of single cells within their entire tissue context, are relatively low through-put, and face unique challenges for isolating protoplasts from plant cells due to cell walls (**Figure 1**). The development of spatial transcriptomics technologies now offers the capability to complete the picture of gene expression in the spatiotemporal context (Giacomello, 2021; Tao et al., 2026).

Spatial transcriptomics has transformed the study of gene expression, enabling transcripts within a tissue section to be quantified and mapped back into the original cell and tissue architecture, at detailed resolution and with high efficiency. A number of spatial transcriptomics platforms have been developed with differing techniques to visualise gene expression. These include *in situ* hybridisation, based on hybridisation of target sequences to fluorescing probes to capture their location within the tissue, *in situ* sequencing, based on sequencing transcripts in place with padlock probes, and *in situ* capture, which captures the entire RNA contents of the tissue with spatially barcoded probes, allowing them to be mapped back into the original tissue structure after sequencing (**Figure 1**) (Deng et al., 2025). The evolution of these technologies, detailed methodological differences, and their benefits and limitations for studying different tissues of interest have been covered in multiple reviews (Deng et al., 2025; Moskal et al., 2025; Shao et al., 2026). In plants, spatial transcriptomics has been used to study *Arabidopsis thaliana* leaves (Xia et al., 2022), grain crops such as barley, maize and wheat, (Peirats-Llobet et al., 2023; Fu et al., 2023; Wang et al., 2024; Li et al., 2025; Millsteed et al., 2025, 2026), and fruits and vegetables (Song et al., 2023; Li et al., 2024; Reddy et al., 2025). Therefore, these techniques have already greatly advanced the knowledge of gene expression in various plant species and tissues. However, some limitations of the technology have been identified, especially when applied to seeds. These include issues capturing genes expressed at very low levels, issues working with cells with high water and starch content and rigid cell walls (a problem for applying spatial transcriptomics to plant tissues), interference of autofluorescence (a problem for methods using fluorescently labelled probes), and challenges analysing polyploid genomes and large data volumes (Shao et al., 2026). Given the agricultural and ecological significance of seeds, improving the capacity to capture detailed and accurate transcriptomics data from them should be a key focus for future plant science research.

While several reviews on spatial transcriptomics for crop improvement have been published (Hu et al., 2024; Barmukh et al, 2025; Shao et al, 2026), there are no reviews to date specifically focusing on applications of this technology to seeds. Here, we conduct a review of spatial transcriptomics studies in seeds, highlighting the major contributions of each to the understanding of seed development and germination, and the technological challenges faced.

## Applications of spatial omics in seeds

### Wheat

Wheat is one of the most agronomically significant crops in the world and a major target of spatial transcriptomics research. Li et al. (2025) first undertook a spatial transcriptomics analysis of the developing wheat grain through 4, 8 and 12 days post anthesis (DPA), using the BMKMANU S1000 *in situ* capture platform. They highlighted 10 key gene expression clusters within the grain structure and uncovered differentially expressed genes involved with regulating grain development. This included the transcription factor TaABI3-B1, which was specifically expressed in the embryo and embryo surrounding region (ESR), and which inversely affects embryo and grain size (**Table 1**). This research was then built upon by Millsteed et al. (2025), using the Stereo-seq *in situ* capture platform to analyse spatial gene expression in the 14 DPA wheat seed. This study also delineated 8 major gene expression clusters, including four distinct endosperm clusters with unique expression profiles linked to starch and protein biosynthesis and lipid transfer activity. These gene expression clusters largely aligned with the findings from Li et al. (2025) and give new detail about the spatial progression of starch and protein deposition from the inner to outer endosperm during grain filling. Additionally, the expression patterns of some grain quality genes were investigated, highlighting subgenome biased expression for puroindoline-B and alpha-amylase inhibitor (**Table 1**). Recently, Fan et al. (2026) published a transcriptomics atlas of the wheat grain at the milk stage of development (25 DPA), integrated with single nucleus (sn)RNA-seq data. This study used the BMKMANU S1000 platform to identify 13 transcriptionally and spatially distinct cell clusters, as well as notable dominance of subgenome B expression. Lastly, the authors highlighted that the NAC transcription factors are key regulators of storage metabolism, grain maturation, and programmed cell death (**Table 1**). Here, combining the spatial data with snRNA-seq allowed for comprehensive cell-type annotation (Fan et al., 2026).

**Table 1:** Overview of spatial transcriptomics research on seeds including the species studied, the capture type, developmental/germination stage, and major contributions to the understanding of seed structure, development and germination made by the referenced studies.

Species	Technology	Development/ germination stage	Main contributions	Reference
Wheat	BMKMANU S1000 (*In situ* capture)	4, 8, 12 DPA	- 10 gene expression clusters identified - Transcription factor TaABI3-B1 inversely affects grain size	Li et al., 2025
	Stereo-seq (*In situ* capture)	14 DPA	- 8 gene expression clusters in concentric pattern identified - Subgenome biased expression of quality genes	Millsteed et al., 2025
	BMKMANU S1000	25 DPA	- 13 gene expression clusters - Dominance of subgenome B - NAC transcription factors control programmed cell death and seed maturation	Fan et al., 2026
Barley	10x Genomics Visium (*In situ* capture)	0, 1, 3, 6, 24 HAI	- Process of germination is highly spatially organised - >14,000 differentially expressed genes in first 24h HAI	Peirats-Llobet et al., 2023
	10x Genomics Visium	3, 6, 12, 20 DPA	- First spatial study to follow seed development and germination - Quantitative transcript abundance of >20,000 genes	Peirats-Llobet et al., 2026
Maize	10x Genomics Visium	10, 14, 18DPA	- Distinct spatial expression of starch biosynthesis, and protein and lipid storage genes - Distinct spatial expression of seven different sucrose transporters	Fu et al., 2023
Rice	Stereo-seq and scRNA-seq	6, 24, 36, 48 HAI	- Two new scutellum cell types characterised - Development of automated cell segmentation protocol	Yao et al., 2024
Wild soybean	10x Genomics Visium and scRNA-seq	Mid-maturity stage	- Distinct spatial patterns of nutrient accumulation into the adaxial and abaxial regions - Gene GsMAPK23–4 is involved with protein accumulation	Liu et al., 2026
Arabidopsis	Curio Seeker (*In situ* capture), MERSCOPE (*in situ* hybridisation) and snRNA-seq	0, 1.25 DAI (germination) and 47 DAI (developing seeds in siliques)	- Spatial expression patterns of flavonoid biosynthesis genes in seed coat and endosperm - Comprehensive transcriptomics atlas for Arabidopsis lifecycle	Lee et al., 2025

Spatial transcriptomics has advanced the knowledge of wheat seed development by providing the spatial context for genes controlling grain size, protein and starch deposition, and programmed cell death. This new spatial detail explains how coordinated gene expression between tissues drives grain formation and constructs the physical and functional architecture of the grain, through to maturation. The insights gained from spatial transcriptomics offer a valuable framework for next generation crop improvement; however, some challenges were also identified in applying the technology to seeds. Most notably, the ability to capture genes expressed at very low levels during early development was limited (Li et al., 2025), the capture chip was not optimised for the developing wheat grain tissue, resulting in the tissue edges lifting and limiting transcript localisation of some cell regions (Millsteed et al., 2025), and the analysis pipeline for polyploid genomes requires further streamlining. For example, the difference in number of clusters identified was likely a technical artifact due to the different capture efficiencies of the technologies or the different clustering methods used for analysis. Therefore, this field would benefit from standardised analysis steps, such as the types of normalisation and clustering methods used, and further optimisation of the *in situ* capture steps.

### Barley

A spatial transcriptomics analysis of the germinating barley grain was first conducted by Peirats-Llobet et al. (2023) using the 10x Genomics Visium *in situ* capture platform. This study assessed spatial gene expression over the first 24 hours of germination at 0, 1, 3, 6 and 24 hours after imbibition (HAI), and revealed highly organised patterns of gene expression across the main seed tissues, the embryo, aleurone and endosperm. The embryo exhibited high expression of genes linked to transcription, translation, elongation factors and heat shock factors. The aleurone and endosperm were primarily characterised by transcripts associated with nutrient storage, starch degradation and metabolism functions, including late embryogenesis abundant (LEA) proteins, gamma-gliadins, alpha-amylase, alpha-amylase inhibitors, trypsin inhibitors, and the aquaporin gene family. Over 14,000 genes were found to be differentially expressed across tissue types throughout the first 24 HAI, providing new insight about how nutrient use and water uptake are spatiotemporally regulated to support germination (**Table 1**) (Peirats-Llobet et al., 2023). This was followed by a spatial transcriptomics analysis of the developing barley grain at 3, 6, 12 and 20 DPA, combined with the spatial germination data, to create a comprehensive transcriptomics atlas (Peirats-Llobet et al., 2026). This study provided quantitative transcript abundance of over 20,000 genes and identified heterogeneous expression across spatial domains for numerous biological processes in the seed lifecycle (**Table 1**). Though the authors noted that the capture spot size limited the ability to obtain single cell resolution of the barley grain cells, this was the first spatial analysis of seeds to follow both the processes of development and germination and represents a valuable resource for future targeted genetic research of barley (Peirats-Llobet et al., 2026).

### Maize

The maize kernel was analysed by Fu et al. (2023) using the 10x Genomics Visium platform, focusing on the process of sucrose transport and distribution after delivery from the phloem at 10, 14, and 18 DPA. By mapping gene expression across kernel tissues, the authors identified 25 spatially distinct cell clusters that, when aligned with the anatomical images of the tissue, were designated into 11 functional cell populations. Many genes encoding starch biosynthesis enzymes were identified as marker genes for the starchy endosperm, including sucrose synthase, glucose-1-phosphate adenylyltransferase large and small subunits, adenine nucleotide transporter (BT1), and starch synthases 1 and 2. Protein storage genes were also highly expressed in the endosperm, while most of the oil and lipid storage genes were expressed in the embryo. Seven sucrose transporter genes were identified in the maize genome (*ZmSUT1*, *ZmSUT2*, *ZmSUT3*, *ZmSUT4*, *ZmSUT5*, *ZmSUT6*, and *ZmSUT7*), while two (*ZmSUT1* and *ZmSUT7*) were found to have specific spatial expression in the basal endosperm transfer layer (BETL), where they likely play a key role in post-phloem sucrose transport into the endosperm (**Table 1**). The spatial transcriptomics data showed that transporter genes are expressed in precise domains, linking sucrose unloading to starch biosynthesis and kernel nutritional quality. These spatial gradients of sugar metabolism explain how nutrient allocation shapes kernel composition in greater detail than could previously be obtained through bulk or single cell transcriptomics studies. However, this study also identified a limitation of the spatial resolution of the technology, as each RNA capture spot may represent transcripts from multiple cells (Fu et al., 2023), meaning that the fine-scale single-cellular resolution could still be further enhanced.

### Rice

The germinating rice seed was analysed using both Stereo-seq and single-cell (sc)RNA-seq, at 6, 24, 36 and 48 HAI, to cover the rapid water uptake phase and the metabolism and mobilisation (lag) phase of germination, as these represent periods of significant transcriptional change (Yao et al., 2024). The authors generated a high-resolution atlas of the germinating embryo, identifying 14 distinct embryonic cell types, including two previously uncharacterised scutellum cell types (**Table 1**). This study highlighted distinct changes in cell composition, with radicle-associated cells increasing and some scutellum cells decreasing over time. Overall, gene expression transitioned from early stress-response and environmental signalling pathways to later activation of the cell cycle, DNA replication, and growth-related processes. Additionally, hormonal regulation was spatiotemporally resolved, with increasing auxin and gibberellin activity observed alongside decreasing abscisic acid signalling, likely in preparation for nutrient mobilisation. The major contribution of this study was combining the use of Stereo-seq and scRNA-seq to build a detailed spatiotemporal atlas of rice seed germination and develop an automated cell segmentation method based on deep learning. While this analysis was able to delineate new detail about gene expression patterns during rice germination, some challenges with the methodology were also noted. The experiment omitted the time point of 0 HAI as the dry seeds at this time were too brittle to cut sections. Additionally, there were limitations in identifying some cell types at the other time points studied due to the cutting angle of the sections obtained (Yao et al., 2024).

### Wild soybean

A study by Liu et al. (2026) integrated high-throughput spatial transcriptomics (10 x Genomics Visium platform), spatial metabolomics, and scRNA-seq to undertake a multi-omics analysis of the developing wild soybean (*Glycine soja)* seed at the mid-maturity stage. This study generated a high-resolution map of gene expression and metabolite distribution, showing distinct spatial patterns in nutrient accumulation. Lipid biosynthesis pathways were predominantly enriched in adaxial cell regions, while protein and amino acid metabolism were more active in abaxial regions. Distinct transcriptional programs were identified across the different cell types, highlighting the coordinated regulatory networks that drive seed filling. The study also showed evidence of cell–cell communication through ligand-receptor analysis, whereby the seed coat cells act as regulatory hubs in communication networks. Lastly, a key regulatory gene, GsMAPK23-4, was identified as a negative regulator of protein and amino acid accumulation in the cotyledon, with knockout mutants showing significantly higher levels of amino acids and proteins (**Table 1**). By combining multi-omics techniques, this study offered several new insights about soybean seed development that were not previously accessible, moving beyond a simple stage-based model of development to understanding the spatial patterns of cell-cell communication and cell-state changes taking place. This also allowed for metabolites to be linked to discrete tissue regions, highlighting how nutrient accumulation is spatially regulated. Despite these insights, there were some methodological limitations, notably that the analysis was restricted to a single developmental time point. Additionally, this scRNA-seq method lacked the resolution to clearly annotate the boundaries between all cell types identified, though this was aided by comparison with the spatial transcriptomics data (Liu et al., 2026).

### Arabidopsis

The transcriptome of the entire Arabidopsis lifecycle was studied by Lee et al. (2025), using a combination of spatial transcriptomics and snRNA-seq. The resulting transcriptomics atlas covered developmental stages from seeds at 0 and 1.25 days after imbibition (DAI) through to developing seed sacs within the silique at 47 DAI, and was achieved using the Curio Seeker *in situ* capture based technology (for the germinating seeds) and MERSCOPE *in situ* hybridisation technology (for the siliques). This study generated a wealth of data about Arabidopsis seed function, but specifically highlighted the expression of genes associated with flavonoid biosynthesis pathways in both the seed coat and endosperm of developing seeds. While flavonoid secondary metabolites are known to be involved with determining seed coat colour, for example the transparent testa phenotype in mutants of flavonoid biosynthetic enzymes (Appelhagen et al., 2014), the expression of these genes in the endosperm may be linked to regulating fatty acid levels in the embryo (Xuan et al., 2018). The distinct spatial expression patterns of these genes points to functionally diverse roles of flavonoid metabolites between discrete cell types within the seed (**Table 1**). Here, the combination of spatial transcriptomics and snRNA-seq data has advanced the knowledge of gene expression in Arabidopsis, demonstrating that transcriptional pathways may be associated with secondary metabolite production, across multiple cell types. However, the level of influence of secondary metabolite pathways throughout the Arabidopsis lifecycle represents an area for further research. Lastly, it was noted that some of the samples studied lacked comprehensively annotated reference datasets (Lee et al., 2025). Access to comprehensive reference datasets would significantly improve the scope of spatial transcriptomics research in the future.

## Challenges and future directions

Advances in spatial transcriptomics technologies have greatly increased understanding of the mechanisms behind seed development and germination. These technologies have provided the capacity to map spatiotemporal regulatory pathways with detailed, fine-scale resolution, that couldn’t previously be achieved. Some of the major contributions of spatial transcriptomics studies in seeds are the characterisation of novel cell populations and cell-subtypes (Yao et al., 2024), identification of gene expression gradients across developmental stages and tissue types (Li et al., 2025; Millsteed et al., 2025), and explaining how gene expression within different tissues dictates the physical structure of the seed and its functional and nutritional properties (Fu et al., 2023; Liu et al., 2026). Spatial transcriptomics has revealed new detail about the spatial progression of grain filling and embryogenesis, through to the spatiotemporal regulation of water and nutrient use to support germination, adding to the knowledge about seed development and the beginning of the new plant’s lifecycle. This greater detail will facilitate more targeted genetic research, starting from the seed to benefit agriculture and ecological conservation efforts.

However, technological challenges with applying spatial omics to seeds have also been identified. For example, it is currently not possible to achieve true single cell resolution at all developmental stages and in all cell types in seeds. The capture spot size of the 10x Genomics Visium platform could not facilitate true single cell resolution due to the varying sizes of the plant cells analysed, resulting in capture spots representing transcripts from multiple cells in some areas of the tissue (Fu et al., 2023). Additionally, the BMKMANU S1000 platform had reduced capture efficiency of low expressed genes during very early embryo development (Li et al., 2025), and the Stereo-seq platform capture chip surface was not optimised for mounting the wheat seed tissue (likely due to the higher starch and water content of the seed compared to mammalian systems), resulting in the tissue edges lifting and limiting transcript localisation of some cell regions (Millsteed et al., 2025). Therefore, the capture chip for these *in situ* capture platforms require optimisation of the spot size, probe design and surface material or coating for more stringent single cell resolution in seeds. In addition, seed specific tissue preparation protocols could help address issues with capturing transcripts from seeds. Interestingly, nearly all spatial transcriptomics of seeds studies to date have used platforms based on *in situ* capture techniques. This may be due to the ability to sequence all transcripts present in the sample with a relatively streamlined workflow, rather than being limited to a predesigned panel of target genes as with *in situ* hybridisation, or undertaking more complicated workflows and data analysis as with *in situ* sequencing (Shao et al., 2026). Additionally, *in situ* hybridisation platforms depend on fluorescing probes to detect the locations of target sequences, which may be obscured by the natural autofluorescence of the seed cell walls, particularly the rigid pericarp cell walls (Shao et al., 2026). Therefore, fine-tuning the established *in situ* capture protocols may be the most efficient path to addressing the current gaps in the field.

There are also practical limitations to be addressed. Lack of standardised analysis pipelines for polyploid species, availability of fully annotated reference genomes and computational capacity for large data volumes represent challenges for spatial transcriptomics in seeds (Moskal et al., 2025; Lee et al., 2025; Shao et al., 2026). Therefore, generation of more high-quality annotated reference genomes for important crop species would improve spatial mapping. Additionally, development of a more streamlined bioinformatics pipeline for analysis of polyploid genomes, that can be standardised across spatial transcriptomics data types, would be a significant advance in the efficiency and accuracy of this research. Lastly, the cost of spatial transcriptomics is not accessible to most labs and is a major limiting factor for the scope of the experiments undertaken, meaning that the number of tissue sections, cutting orientations and developmental time points included in the studies so far have been fairly conservative (Peirats-Llobet et al., 2023; Yao et al., 2024; Liu et al., 2026). Improving the accessibility of the cost of these platforms would allow for more detailed experiments, analysis of multiple cutting orientations and time points, and greater contribution from the wider scientific community. Given the agricultural and ecological significance of seeds and that spatial transcriptomics platforms continue to evolve, embedding optimisation for the application in seeds should be a major focus for the future of plant science research. With the ability to link gene expression location to seed traits such as structural development, nutritional composition and germination, it is clear that spatial transcriptomics is the new basis for next generation crop improvement.

## References

[B1] Appelhagen I. Thiedig K. Nordholt N. Schmidt N. Huep G. Sagasser M. . (2014). Update on transparent testa mutants from Arabidopsis thaliana: characterisation of new alleles from an isogenic collection. Planta 240, 955–970. doi: 10.1007/s00425-014-2088-0 24903359

[B2] Barmukh R. Garg V. Liu H. Chitikineni A. Xin L. Henry R. . (2025). Spatialomics for accelerating plant research and crop improvement. Trends Biotechnol. 43, 1904–1920. doi: 10.1016/j.tibtech.2025.03.007 40221306

[B4] Chaudhury A. M. Koltunow A. Payne T. Luo M. Tucker M. R. Dennis E. . (2001). Control of early seed development. Annu. Rev. Cell Dev. Biol. 17, 677–699. doi: 10.1146/annurev.cellbio.17.1.677 11687501

[B5] Deng P. Huang J. He W. Li Z. Guo C. Chen G. . (2025). Opportunities and challenges in the application of spatiotemporal transcriptomics in plant research. Front. Plant Sci. 16. doi: 10.3389/fpls.2025.1684057 41169728 PMC12568615

[B6] Dwivedi S. L. Spillane C. Lopez F. Ayele B. T. Ortiz R. (2021). First the seed: Genomic advances in seed science for improved crop productivity and food security. Crop Sci. 61, 1501–1526. doi: 10.1002/csc2.20402 41531421

[B7] Fan J. Xue Q. Li J. Liu Y. Liu F. Li Z. . (2026). Single-cell and spatial transcriptomics reveal the regulatory continuum from initial substrates to storage compounds during wheat grain development. Plant Physiol. Biochem. 235, 111376. doi: 10.1016/j.plaphy.2026.111376 42160834

[B8] Fu Y. Xiao W. Tian L. Guo L. Ma G. Ji C. . (2023). Spatial transcriptomics uncover sucrose post-phloem transport during maize kernel development. Nat. Commun. 14. doi: 10.1038/s41467-023-43006-7 37938556 PMC10632454

[B9] Giacomello S. (2021). A new era for plant science: spatial single-cell transcriptomics. Curr. Opin. Plant Biol. 60, 102041. doi: 10.1016/j.pbi.2021.102041 33915520

[B10] Hu Y. Dash L. May G. Sardesai N. Deschamps S. (2024). Harnessing single-cell and spatial transcriptomics for crop improvement. Plants 13, 3476. doi: 10.3390/plants13243476 39771174 PMC11728591

[B11] Lee T. A. Illouz-Eliaz N. Nobori T. Xu J. Jow B. Nery J. R. . (2025). A single-cell, spatial transcriptomic atlas of the arabidopsis life cycle. Nat. Plants 11, 1960–1975. doi: 10.1038/s41477-025-02072-z 40830271 PMC12416547

[B12] Li X. Li B. Gu S. Pang X. Mason P. Yuan J. . (2024). Single-cell and spatial rna sequencing reveal the spatiotemporal trajectories of fruit senescence. Nat. Commun. 15. doi: 10.1038/s41467-024-47329-x 38600080 PMC11006883

[B13] Li X. Wan Y. Wang D. Li X. Wu J. Xiao J. . (2025). Spatiotemporal transcriptomics reveals key gene regulation for grain yield and quality in wheat. Genome Biol. 26. doi: 10.1186/s13059-025-03569-8 40217326 PMC11992740

[B14] Liu P. Li M. Ma P. Yan H. Liu C. Hu Z. . (2026). Spatiotemporal transcriptomic and metabolomic landscapes of wild soybean seed development reveal regulatory mechanisms of nutrient accumulation. Plant Commun. 7, 101580. doi: 10.1016/j.xplc.2025.101580 41169047 PMC12902272

[B15] Millsteed T. Kainer D. Sullivan R. Sun X. Li K. L. Mao L. . (2025). Spatial transcriptomics of developing wheat seed reveals concentric gene expression zones and subgenome biased expression of key genes. Plant Biotechnol. J. 23 (12). doi: 10.1111/pbi.70351 40904198 PMC12665067

[B16] Millsteed T. Kainer D. Sullivan R. Sun X. Li K. L. Mao L. . (2026). Going against the grain: Investigating C4 photosynthesis in the wheat grain with spatial transcriptomics. BMC Plant Biol. doi: 10.1186/s12870-026-09008-5 42192524 PMC13393771

[B18] Moskal K. Puchta-Jasinska M. Bolc P. Motor A. Frankowski R. Pietrusinska-Radzio A. . (2025). Why “where” matters as much as “how much”: Single-cell and spatial transcriptomics in plants. Int. J. Mol. Sci. 26, 11819. doi: 10.3390/ijms262411819 41465249 PMC12732828

[B17] Nowack M. K. Ungru A. Bjerkan K. N. Grini P. E. Schnittger A. (2010). Reproductive cross-talk: seed development in flowering plants. Biochem. Soc Trans. 38, 604–612. doi: 10.1042/BST0380604 20298229

[B20] Peirats-Llobet M. Staka Z. Yang X. Zhu Y. He C. Hurgobin B. . (2026). A four-dimensional spatial transcriptome atlas of barley caryopsis development and germination. Plant Cell 38, koag120. doi: 10.1093/plcell/koag120 42015541 PMC13213456

[B19] Peirats-Llobet M. Yi C. Liew L. C. Berkowitz O. Narsai R. Lewsey M. G. . (2023). Spatially resolved transcriptomic analysis of the germinating barley grain. Nucleic Acids Res. 51, 7798–7819. doi: 10.1093/nar/gkad521 37351575 PMC10450182

[B21] Reddy U. K. Karnatam K. S. Talavera-Caro A. Lopez-Ortiz C. Ku K.-M. Chinreddy S. R. . (2025). Uncovering the genetic architecture of pungency, carotenoids, and flavor in capsicum chinense via twas-mgwas integration and spatial transcriptomics. Hortic. Res. 12. doi: 10.1093/hr/uhaf243 41393923 PMC12701575

[B22] Shao W. Sitepu I. Guo X. Qian B. Wei T. Sahu S. K. (2026). Decoding spatial heterogeneity in plants: Advances and challenges in spatial transcriptomics for stress responses and crop resilience. Plant Stress 20, 101300. doi: 10.1016/j.stress.2026.101300 38826717

[B23] Shewry P. R. Mitchell R. A. Tosi P. Wan Y. Underwood C. Lovegrove A. . (2012). An integrated study of grain development of wheat (cv. hereward). J. Cereal Sci. 56, 21–30. doi: 10.1016/j.jcs.2011.11.007 38826717

[B24] Song X. Guo P. Xia K. Wang M. Liu Y. Chen L. . (2023). Spatial transcriptomics reveals light-induced chlorenchyma cells involved in promoting shoot regeneration in tomato callus. Proc. Natl. Acad. Sci. 120. doi: 10.1073/pnas.2310163120 37703282 PMC10515167

[B25] Tao X.-Y. Tan C. Liu Y. Wang Y. Raza A. He J. . (2026). The potential of wheat spatial omics. Nat. Genet. 58. doi: 10.1038/s41588-026-02542-w 41922865

[B26] Theissinger K. Fernandes C. Formenti G. Bista I. Berg P. R. Bleidorn C. . (2023). How genomics can help biodiversity conservation. Trends Genet. 39, 545–559. doi: 10.1016/j.tig.2023.01.005 36801111

[B27] Wang Y. Luo Y. Guo X. Li Y. Yan J. Shao W. . (2024). A spatial transcriptome map of the developing maize ear. Nat. Plants 10, 815–827. doi: 10.1038/s41477-024-01683-2 38745100

[B28] Xia K. Sun H.-X. Li J. Li J. Zhao Y. Chen L. . (2022). The single-cell stereo-seq reveals region-specific cell subtypes and transcriptome profiling in arabidopsis leaves. Dev. Cell 57, 1299–1310.e4. doi: 10.1016/j.devcel.2022.04.011 35512702

[B29] Xuan L. Zhang C. Yan T. Wu D. Hussain N. Li Z. . (2018). TRANSPARENT TESTA 4-mediated flavonoids negatively affect embryonic fatty acid biosynthesis in Arabidopsis. Plant Cell Environ. 41, 2773–2790. doi: 10.1111/pce.13402 29981254

[B30] Yao J. Chu Q. Guo X. Shao W. Shang N. Luo K. . (2024). Spatiotemporal transcriptomic landscape of rice embryonic cells during seed germination. Dev. Cell 59, 2320–2332.e5. doi: 10.1016/j.devcel.2024.05.016 38848718

